# Clustering students into groups according to their learning style

**DOI:** 10.1016/j.mex.2019.09.026

**Published:** 2019-09-24

**Authors:** Irene Pasina, Goze Bayram, Wafa Labib, Abdelhakim Abdelhadi, Mohammad Nurunnabi

**Affiliations:** a College of Engineering, Prince Sultan University, P.O. Box 66833, Riyadh 11586, Saudi Arabia; b College of Business Administration, Prince Sultan University, P.O. Box 66833, Riyadh 11586, Saudi Arabia; c St Antony’s College, University of Oxford, 62 Woodstock Road, Oxford OX2 6JF, UK

**Keywords:** Hierarchal clustering algorithms, Learning style, Group technology, Felder and Silverman, Teaching style

## Abstract

This method article aims to use group technology to classify engineering students at classroom level into clusters according to their learning style preferences. The Felder and Silverman’s Index Learning Style (ILS) was used to evaluate students’ learning style preferences. Students were then grouped into clusters based on the similarities of their learning styles preferences by using clustering algorithms, such as complete clustering.

• Prior research on Learning Styles preferences in engineering education is limited in Saudi Arabia.
• Students’ learning style preferences allows instructors to adopt suitable teaching approach. Students having same learning styles can work together in group assignments.
• Grouping students into clusters, we find that outlier students who having different learning styles than the rest may allow instructors to deal with them accordingly.

## Graphical abstract

**Figure:**
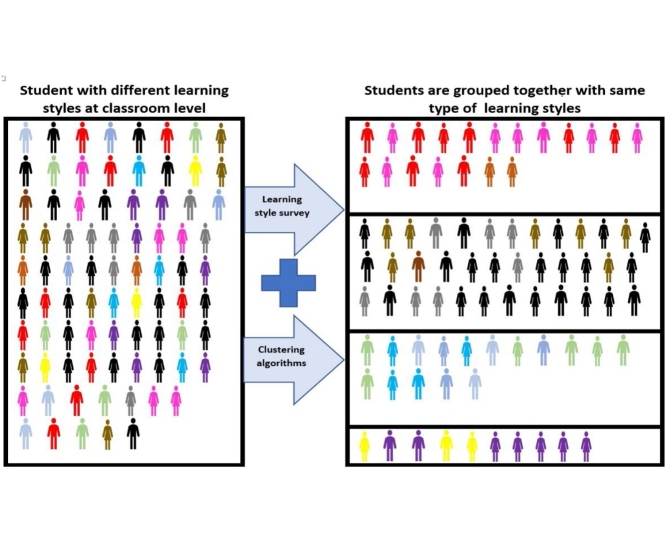

**Specification Table**

**Table:** 

Subject Area:	Engineering
More specific subject area:	Higher Education - Engineering
Method name:	Hierarchal Clustering Algorithms
Name and reference of original method:	Felder, R. M. (1996). Matters of style. ASEE Prism, 6(4), 18-23. http://www4.ncsu.edu/unity/lockers/users/f/felder/public/Papers/LS-Prism.htm (accessed 23 Feb 2019). (Appendix A)
Resource availability:	The data is available in the article

## Data and method details

Students’ learning style and their learning preferences have an impact on their learning achievement. For this reason, they have been investigated by researchers, who considered a variety of factors like gender, level and type of education [[1], [2], [3], [4]]. Knowing the students’ learning style preferences allows instructors in higher education to adopt suitable teaching approach to improve the students’ academic performance [[1], [2], [3]]. Bachman [5] explains that students have better performances whenever they work by group based on similar learning styles. Massey and al. suggest that this can be beneficial also for the instructors, as they can implement the learning opportunities in their courses analyzing the students’ experience [6], while Nielsen and Kreiner [7] propose that students’ learning styles could be considered in the course evaluation. Knowing the students’ learning styles allows instructors to select the correct teaching technique [8], as one of the major difficulties that college’ teachers face is to understand how to enhance academic achievements by combining the instruction techniques and the students’ learning style [9]. When instructors plan the learning contents and assignments in their course, they should consider the differences in learning style between the students, has Haden indicates [10]. Giles and al. [11] show another approach, using statistically controlled design to analyze the impact of teaching style on learning and, consequently, to assess these approaches in quantitative courses, and establish protocols for such studies.

This study examines the use group technology to classify engineering students at classroom level into clusters according to their learning style preferences in Saudi Arabia using the Index of Learning Styles (ILS) developed by Felder [[1], [2], [3]].

## Learning styles

Several learning-style models have been used to examine and testing in Engineering education [2,3]. In this study, we adopted the classification developed by Felder and Silverman, which consisted in 44 questions referring to four dimensions: The reasons for choosing this Index Learning Style (ILS) were based on the wider acceptability of the model [[3], [4], [5], [6], [7]].

• What type of information the student preferentially perceives: sensory, SEN or intuitive, INT?
• What type of sensory information is most effectively perceived: visual, VIS or verbal, VRB?
• How students prefer to process the information: actively, ACT or reflectively, REF.
• How the student characteristically progresses toward understanding: sequentially, SEQ or globally, GLO.

We conducted a systematic literature review which revealed that students’ learning style preferences have been investigated through the use of descriptive statistical analyses (e.g. frequencies and percentages of the targeted population). Prior studies also reported the preferences of the targeted student populations individually. The Index Learning Style (ILS) developed by Richard Felder and Barbra Nancy in 1991 comprises 44 questions to assess preferences on four sets of responses. This questionnaire was designed to find out what you learning preferences are. It was originally designed by Felder and Silverman at North Carolina State University, USA. Students are instructed to complete the questionnaire and select "a" or "b" to indicate answer to every question. Students choose one answer for each question and answer every question mandatorily. If both "a" and "b" seem to apply, student choose the one that applies more frequently.

The ILS is available free-of-charge from the World Wide Web to individuals who would like to assess their own preferences. It is obvious that the studies done on this subject used descriptive analyses, and they described the types of learners accordingly. Based on a student’s learning style, the instructor has to use proper approaches of teaching. For example, if a student belongs to sensing/active types of learning, a balanced set of material that emphasizes practical problem-solving methods needs to be presented with material that emphasizes fundamental understanding for intuitive/reflective students [12,13].

The current approach to learning style deals with the majority of students and the type of learners that they belong to in order to use a suitable method for teaching students and helping the outlier students whom are lagging. Surveying United Arab Emirates Universities undergraduate statistics students, Darwish [14] concluded that students preferred reflective over active, intuitive over sensing, verbal over visual, and global over sequential learning styles.

This study intends to divide students into clusters having common learning styles in order for the educator to differentiate between students learning capabilities in assessments and accordingly using a suitable teaching style to present the class materials. This approach can also find the outlier students and deal with them accordingly.

The similarity coefficient is used to identify the relationship between parts with regard to certain characteristics under invistigation. Based on this relationship, groups of items are identified. Among the algorithms that are used to identify and to form part-families that are associated with the machine cell formation, clustering algorithms are based on the similarity coefficient method, which is used to find similarities between parts/machines, and then to group them into part-families/machine cells. Pairwise similarity coefficients between machines/parts are calculated by using specific similarity coefficient formulas. These similarities are then organized into a matrix called the similarity coefficient matrix. This matrix is used as an input for one of the clustering algorithms, such as single-linkage clustering (SLINK), complete linkage clustering, or average linkage clustering, to form part-families/machine cells, where the inputs can be the distances or similarities between pairs of objects. Single-linkage clustering forms groups by merging the nearest neighbors together according to the highest similarities between them [15] . It works as follows:

• Start with M cluster containing a M × M symmetric matrix of distance/similarities in $\text{D}=\left\{{\text{d}}_{\text{i}\text{k}}\right\}$
• Find the smallest distances/similarities in $\text{D}=\left\{{\text{d}}_{\text{i}\text{k}}\right\}$
• Merge the correspong objects, U and V, to obtain the cluster UV
• The distance/similarities between UV and any other cluster, Q, is computed by:

(1) $$D_{UVQ}\;=\;min\left\{d_{UQ},d_{VQ}\right\}$$

The values d_UQ_ and d_VQ_ are the distances/similarities between the clusters U and Q, and V and Q, respectively. The result is graphically shown in the form of a tree diagram (dendrogram). The tree diagram representing the machine cells/part-families at different levels of similarity is created by using the similarity coefficient matrix.

## Experimental design and discussion

Five instructors were involved in this study to collect data; the instructors explained to volunteer students about the purpose and the importance of different learning styles. More emphasis was given to how the study would reflect on the teaching style and approach of the course assessments. Students were given a link to answer the ILS questions. The responses were then collected for data analysis. Regarding research ethics, informed consent was signed by all participants, and guaranteed about the confidentiality of the data, and that no personal information would be used, such as students’ names.

After conducting the survey, each student receives the results expressed in terms of the scores for different learning-style dimensions (ACT/REF, SEN/INT, VIS/VRB and SEQ/GLO).

• A score of 1 or 3 in any dimension means the student is fairly balanced on the two categories of the dimension,
• A score of 5 or 7 means that the student has a moderate preference in one of the categories of that dimension.
• A score of 9 or 11 means that the student has a strong preference for one category of that dimension and may fail to understand the subject if taught in an environment that does not address his style preference [12].

Table 1 shows the results obtained from 72 participants enrolled in an introductory engineering course class.

**Table 1:** Introductory Engineering Students’ Index Learning Style (ILS) results.

Student	1	2	3	4	5	6	7	8	9	10	11	12	13	14	15	16	17	18	19	20	21	22	23	24
Active	5	7	0	5	7	1	0	1	0	3	3	0	3	0	7	0	9	3	1	1	1	1	5	0
Reflective	0	0	3	0	0	0	5	0	1	0	0	3	0	1	0	7	0	0	0	0	0	0	0	7
Sensing	3	5	0	0	0	3	3	3	0	3	3	1	0	1	0	5	3	1	2	3	4	7	0	3
Intuitive	0	0	9	5	7	0	0	0	1	0	0	0	7	0	3	0	0	0	0	0	0	0	5	0
Visual	9	9	7	11	11	11	7	7	7	5	9	1	11	3	3	7	9	3	1	3	4	5	9	7
Verbal	0	0	0	0	0	0	0	0	0	0	0	0	0	0	0	0	0	0	0	0	0	0	0	0
Sequential	1	7	3	3	5	0	5	1	1	0	7	5	0	0	7	3	3	3	3	3	4	1	0	0
Global	0	0	0	0	0	1	0	0	0	3	0	0	3	3	0	0	0	0	0	0	0	0	1	3
																								
Student	25	26	27	28	29	30	31	32	33	34	35	36	37	38	39	40	41	42	43	44	45	46	47	48
Active	3	3	1	5	1	9	0	0	0	0	0	0	0	0	1	3	3	7	0	0	0	5	0	0
Reflective	0	0	0	0	0	0	1	1	1	1	1	3	3	1	0	0	0	0	1	3	3	0	3	3
Sensing	5	5	1	0	0	1	0	0	0	0	0	0	0	9	5	0	0	1	5	5	0	1	3	3
Intuitive	0	0	0	3	5	0	3	2	3	2	3	5	7	0	0	7	3	0	0	0	7	0	0	0
Visual	0	9	3	9	7	7	7	0	0	0	0	7	9	7	9	11	11	3	9	9	0	7	5	7
Verbal	1	0	0	0	0	0	0	0	1	1	1	0	0	0	0	0	0	0	0	0	1	0	0	0
Sequential	3	1	1	0	0	9	1	1	1	1	1	1	0	3	5	0	5	3	0	1	0	3	9	0
Global	0	0	0	1	1	0	0	0	0	0	0	0	1	0	0	3	0	0	1	0	3	0	0	5
																								
Student	49	50	51	52	53	54	55	56	57	58	59	60	61	62	63	64	65	66	67	68	69	70	71	72
Active	3	7	5	5	9	7	0	0	3	9	0	3	0	0	7	1	3	0	5	7	1	5	7	0
Reflective	0	0	0	0	0	0	3	5	0	0	9	0	1	3	0	0	0	1	0	0	0	0	0	3
Sensing	9	3	9	0	9	5	9	5	5	0	0	0	1	3	1	0	5	3	9	5	0	9	5	9
Intuitive	0	0	0	1	0	0	0	0	0	7	7	1	0	0	0	5	0	0	0	0	1	0	0	0
Visual	9	5	3	3	4	1	0	9	5	11	0	7	3	5	3	7	7	5	5	7	3	0	1	3
Verbal	0	0	0	0	0	0	3	0	0	0	5	0	0	0	0	0	0	0	0	0	0	3	0	0
Sequential	5	1	3	0	3	3	0	2	5	0	0	3	5	5	3	0	3	1	3	0	0	3	0	0
Global	0	0	0	1	0	0	3	0	0	7	7	0	0	0	0	1	0	0	0	3	1	0	3	3

**Fig. 1:**
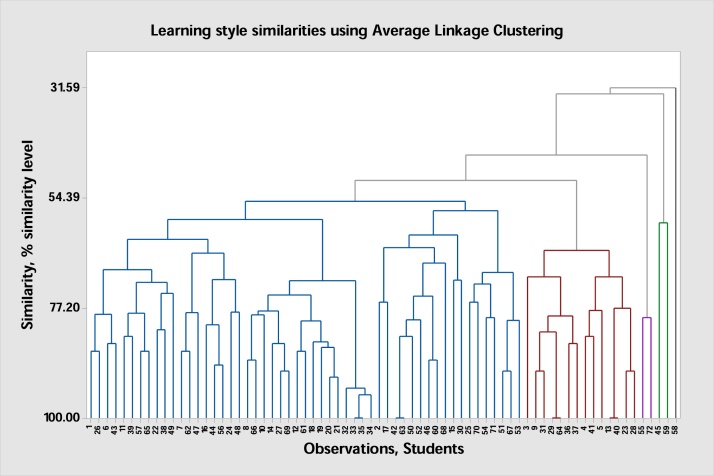
Students are grouped into clusters based on their learning styles.

Fig. 1 presents the results obtained by grouping students into clusters. Average linkage clustering. Studying the results output of the figure, it is clear that students are joining each other in different groups at different level of similarities. For example, the following students joining each other at 100% similarity level between each other (refer to the dendrogram): Student 33 and 35, 29 and 64, 13 and 40. Hence the instructor can ask these students to work together in projects because they can understand each other due to the fact that they are having same learning styles. From the dendrogram, instructor can divide students into groups to work together.

## Conclusion

The findings suggest teaching strategy for instructors. For instance, instructors may use this tool at the beginning of the semester to select appropriate teaching style based on students’ learning styles as groups to achieve the intended teaching outcomes. The results of this study will also reveal teachers explore the learning styles of their students to choose the proper teaching methodology/strategy and assessment method that fits each student group instead of dealing with the classroom as one unit. This helps students to better achieve the intended learning outcomes of the course. Teachers can find outlier students and try to deal with them differently in order to achieve the learning outcomes, for example, in the dendrogram, student number 58 is considered as an outlier and should have special approach due to his deferent learning style from the rest of students.

## Declaration of Competing Interest

The authors declare no conflict of interest.

## Appendix A Index of Learning Styles (ILS) Questionnaire

[To complete the questionnaire please circle "a" or "b" to indicate your answer to every question. You may only choose one answer for each question and you must answer every question. If both "a" and "b" seem to apply to you, please choose the one that applies more frequently]

1 I understand something better after I
  (a) try it out.
  (b) think it through.
2 I would rather be considered
  (a) realistic.
  (b) innovative.
3 When I think about what I did yesterday, I am most likely to get
  (a) a picture.
  (b) words.
4 I tend to
  (a) understand details of a subject but may be fuzzy about its overall structure.
  (b) understand the overall structure but may be fuzzy about details.
5 When I am learning something new, it helps me to
  (a) talk about it.
  (b) think about it.
6 If I were a teacher, I would rather teach a course
  (a) that deals with facts and real life situations.
  (b) that deals with ideas and theories.
7 I prefer to get new information in
  (a) pictures, diagrams, graphs, or maps.
  (b) written directions or verbal information.
8 Once I understand
  (a) all the parts, I understand the whole thing.
  (b) the whole thing, I see how the parts fit.
9 In a study group working on difficult material, I am more likely to
  (a) jump in and contribute ideas.
  (b) sit back and listen.
10 I find it easier
  (a) to learn facts.
  (b) to learn concepts.
11 In a book with lots of pictures and charts, I am likely to
  (a) look over the pictures and charts carefully.
  (b) focus on the written text.
12 When I solve maths problems
  (a) I usually work my way to the solutions one step at a time.
  (b) I often just see the solutions but then have to struggle to figure out the steps to get to them.
13 In classes I have taken
  (a) I have usually got to know many of the students.
  (b) I have rarely got to know many of the students.
14 In reading non-fiction, I prefer
  (a) something that teaches me new facts or tells me how to do something.
  (b) something that gives me new ideas to think about.
15 I like teachers
  (a) who put a lot of diagrams on the board.
  (b) who spend a lot of time explaining.
16 When I'm analysing a story or a novel
  (a) I think of the incidents and try to put them together to figure out the themes.
  (b) I just know what the themes are when I finish reading and then I have to go back and find the incidents that demonstrate them.
17 When I start a homework problem, I am more likely to
  (a) start working on the solution immediately.
  (b) try to fully understand the problem first.
18 I prefer the idea of
  (a) certainty.
  (b) theory.
19 I remember best
  (a) what I see.
  (b) what I hear.
20 It is more important to me that an instructor
  (a) lay out the material in clear sequential steps.
  (b) give me an overall picture and relate the material to other subjects.
21 I prefer to study
  (a) in a group.
  (b) alone.
22 I am more likely to be considered
  (a) careful about the details of my work.
  (b) creative about how to do my work.
23 When I get directions to a new place, I prefer
  (a) a map.
  (b) written instructions.
24 I learn
  (a) at a fairly regular pace. If I study hard, I'll "get it."
  (b) in fits and starts. I'll be totally confused and then suddenly it all "clicks."
25 I would rather first
  (a) try things out.
  (b) think about how I'm going to do it.
26 When I am reading for enjoyment, I like writers to
  (a) clearly say what they mean.
  (b) say things in creative, interesting ways.
27 When I see a diagram or sketch in class, I am most likely to remember
  (a) the picture.
  (b) what the instructor said about it.
28 When considering a body of information, I am more likely to
  (a) focus on details and miss the big picture.
  (b) try to understand the big picture before getting into the details.
29 I more easily remember
  (a) something I have done.
  (b) something I have thought a lot about.
30 When I have to perform a task, I prefer to
  (a) master one way of doing it.
  (b) come up with new ways of doing it.
31 When someone is showing me data, I prefer
  (a) charts or graphs.
  (b) text summarizing the results.
32 When writing a paper, I am more likely to
  (a) work on (think about or write) the beginning of the paper and progress forward.
  (b) work on (think about or write) different parts of the paper and then order them.
33 When I have to work on a group project, I first want to
  (a) have a "group brainstorming" where everyone contributes ideas.
  (b) brainstorm individually and then come together as a group to compare ideas.
34 I consider it higher praise to call someone
  (a) sensible.
  (b) imaginative.
35 When I meet people at a party, I am more likely to remember
  (a) what they looked like.
  (b) what they said about themselves.
36 When I am learning a new subject, I prefer to
  (a) stay focused on that subject, learning as much about it as I can.
  (b) try to make connections between that subject and related subjects.
37 I am more likely to be considered
  (a) outgoing.
  (b) reserved.
38 I prefer courses that emphasise
  (a) concrete material (facts, data).
  (b) abstract material (concepts, theories).
39 For entertainment, I would rather
  (a) watch television.
  (b) read a book.
40 Some teachers start their lectures with an outline of what they will cover. Such outlines are
  (a) somewhat helpful to me.
  (b) very helpful to me.
41 The idea of doing homework in groups, with one grade for the entire group,
  (a) appeals to me.
  (b) does not appeal to me.
42 When I am doing long calculations,
  (a) I tend to repeat all my steps and check my work carefully.
  (b) I find checking my work tiresome and have to force myself to do it.
43 I tend to picture places I have been
  (a) easily and fairly accurately.
  (b) with difficulty and without much detail.
44 When solving problems in a group, I would be more likely to
  (a) think of the steps in the solution process.
  (b) think of possible consequences or applications of the solution in a wide range of areas.
